# Dataset for Bitcoin arbitrage in different cryptocurrency exchanges

**DOI:** 10.1016/j.dib.2021.107731

**Published:** 2021-12-20

**Authors:** Rasa Bruzgė, Alfreda Šapkauskienė

**Affiliations:** Faculty of Economics and Business Administration, Vilnius University, Saulėtekio al. 9, Vilnius LT- 10222, Lithuania

**Keywords:** Arbitrage dataset, High-frequency, Bitcoin, Network analysis, Cryptocurrency exchange

## Abstract

Bitcoin market's efficiency and liquidity questions are being comprehensively analyzed in scientific literature. This dataset serves academics for deeper analysis of these topics as well as it gives relevant information for spotting and evaluating risks in the market. Moreover, practitioners can benefit from the dataset and use it to identify patterns in the market, discover potential earning capabilities, and create effective arbitrage trading strategies. This is the first publicly available dataset that provides unique arbitrage data about pairs of cryptocurrency exchanges. The raw dataset was received by the Bitlocus LT, UAB. Using *dplyr, reshape2, plyr* packages in R we transformed dataset to show the amount of arbitrage which could be earned in 13 different cryptocurrency exchanges from 2019-01-01 to 2020-04-01. We used this dataset to create matrices for each day from 2019-01-01 to 2020-04-01 in order to perform network analysis on Bitcoin arbitrage opportunities (Bruzgė and Šapkauskienė [1]). However, this dataset is beneficial for other purposes such as the evaluation of market's seasonality and day of week effects. The dataset provides values in high-frequency intervals but it is possible to convert data to a suitable data format depending on the research question.

**Specifications Table**

**Table:** 

Subject	Economics, Econometrics and Finance
Specific subject area	Cryptocurrency and Fintech
Type of data	Table Graph Image Figure
How the data were acquired	Data were received from the Bitlocus LT, UAB cryptocurrency exchange.
Data format	Raw Analyzed Filtered
Description of data collection	The Bitlocus LT, UAB is performing arbitrage trading and data were collected from their internal database. Using Python it's algorithmically programmed to execute trades in different cryptocurrency exchanges if they satisfies certain conditions. If transaction is executed, then internal database saves that information and it is possible to collect data from that database. We received collected data from Bitlocus LT, UAB about the most important information in that database.
Data source location	Bitlocus LT, UAB, company code 305727615.
Data accessibility	Repository name: Mendeley Direct Data identification number: 10.17632/sghd8vbvbp.2 Direct URL to data: https://doi.org/10.17632/sghd8vbvbp.2 Repository name: Mendeley Direct Data identification number: 10.17632/7vp83m4tvr.1 Direct URL to data: https://doi.org/10.17632/7vp83m4tvr.1
Related research article	R. Bruzgė, A. Šapkauskienė, Network analysis on Bitcoin arbitrage opportunities, North American Journal of Economics and Finance. 59 (2022), 101562. https://doi.org/10.1016/j.najef.2021.101562

**Value of the Data**

• This dataset provides unique high-frequency data which allow to trace irregular market activities over a period of time and could give a better understanding of price behavior and trading activity.
• The dataset is important for researchers to explore the market's efficiency, liquidity problems, to spot and evaluate risks in the market (high amounts of arbitrage indicates risk).
• The dataset may enable investors to identify patterns in the market and to discover potential earning capabilities.
• The dataset may be used to evaluate the market's seasonality, day of week effects.
• The dataset may provide insights to algorithmic traders to create effective arbitrage trading strategies.

## 1 Data Description

We were inspired to analyze arbitrage data after identifying that the existing literature uses potential rather than real arbitrage data [2]. We present unique high-frequency dataset of algorithmic trading. Given that, the dataset contains different time intervals depending on the timestamp when an arbitrage opportunity occurred. Data is not in equal time intervals, so for data comparison, values were converted from high-frequency minute values to daily values meaning that any empty data gaps were removed. Converted dataset with daily values covered information about the sum of all transactions that occurred that day in a specific exchange.

Table 1 gives a sample of the dataset we used in which we can see at what exchanges occurred arbitrage opportunities at 2019-01-01 00:00:31. It was possible to buy Bitcoin at “Exchange 1” and simultaneously sell it at “Exchange 2”. As an example, in the first line, we can see that it was possible to buy at bitstamp and to sell in cexio exchange. After this transaction, it would be earned 49.65 Eur or 1.12% from the investment size.

**Table 1:** Sample of raw data.

ID	Year	Month	Day	time	arb_exch1	arb_exch2	arb_ticker	arb_prc	arb_amount
1	2019	1	1	00:00:31	bitstamp	cexio	BTCEUR	1.12	49.65
2	2019	1	1	00:00:31	bitstamp	therock	BTCEUR	0.30	0.14
3	2019	1	1	00:00:31	bitstamp	exmo	BTCEUR	3.93	1.27
4	2019	1	1	00:00:31	bitstamp	coinfalcon	BTCEUR	0.22	0.06
5	2019	1	1	00:00:31	bitstamp	coindeal	BTCEUR	0.30	2.08
6	2019	1	1	00:00:31	cexio	exmo	BTCEUR	1.83	0.60
7	2019	1	1	00:00:31	kraken	cexio	BTCEUR	1.47	9.48
8	2019	1	1	00:00:31	kraken	therock	BTCEUR	0.64	0.31
9	2019	1	1	00:00:31	kraken	exmo	BTCEUR	4.29	1.38
10	2019	1	1	00:00:31	kraken	coinfalcon	BTCEUR	0.56	0.15

The sample of the raw dataset is given in Table 3. It covers additional information about the quantity which had to be bought to earn arbitrage, the best sell and the best buy prices, the balance of fiat currency in “Exchange 1” and the balance of cryptocurrency in “Exchange 2”. If there was enough fiat currency in “Exchange 1” and enough cryptocurrency in “Exchange 2”, it means that the transaction was successfully executed and given arbitrage amount was earned. This additional information could be used by investors or the ones interested in the profitability of arbitrage investing. However, we used only the data about arbitrage amounts in order to analyse the network of cryptocurrency exchanges’.

Our dataset had 9,799,130 tick-level records of Bitcoin-to-Euro exchange rate starting from 2019-01-01 00:00:31 to 2020-03-30 23:59:48. Data covered information about different cryptocurrency pairs from 18 cryptocurrency exchanges. These pairs contained information about exchanges in which it was possible to buy and sell simultaneously. Each row presented the amount of arbitrage that it was possible to earn if a transaction would have been executed. Some exchanges covered information just for a short period of time (i.e., a week, or a month) so it was removed from further analysis. After detailed graphical analysis with Power BI and statistical analysis with R, the most popular cryptocurrency exchanges were identified. We filtered data and created two different datasets of “Exchange 1” and “Exchange 2”. “Exchange 1” dataset presented arbitrage amounts in exchanges where one was able to successfully buy Bitcoin, and “Exchange 2” presented exchanges where one was able to successfully sell Bitcoin. When an arbitrage opportunity occurred it was important to have Euros in “Exchange 1” that one could simultaneously buy Bitcoin at “Exchange 1” and sell it to “Exchange 2”.

Each exchange captured a different number of values of arbitrage opportunities [1]. Also, data covered the different time frames for every exchange but most of them contained information from 2019-01-01 to 2020-03-30. The minimum amount when the transaction was executed was 0.01 Eur and the maximum amount reached almost 1000 Eur. It was programmed algorithmically not to execute transactions where arbitrage exceeds 1000 Eur. Usually, amounts of arbitrage increases when exchange is dealing with liquidity issues. If the exchange in which one buys Bitcoin is illiquid, it means that there would be no possibility to sell later.

For further network analysis, it was necessary to transform the high-frequency algorithmic trading data. Our methodology followed the one given by Tasca et al. [3], so to facilitate comparison, we had to convert minute values to daily values. Based on the daily values of “Exchange 1” and “Exchange 2”, we created 455 daily matrices for each day from January 2019 to April 2020 in order to show the amount of arbitrage that was possible to earn by taking every arbitrage opportunity in each exchange. We created matrices using dplyr, reshape2, and plyr packages in R.

Table 2 gives an example of one of 455 daily matrices. First column shows names of exchanges in which it was possible to buy Bitcoin and simultaneously sell it to exchanges’ given in other columns. For the day of 2019-01-13 we can see that most chances to successfully sell Bitcoin was in cexio and exmo exchanges. To be more precise, if Bitcoin was bought in kraken, dsx, bitmarketlt, coindeal, bitlish, bitstamp and coinfalcon exchanges, it could be simultaneously sold to cexio exchange. If there was enough fiat Euro currency in kraken and enought Bitcoin cryptocurrency in cexio exchange, it means that transactions can be executed by earning 292.25 Eur as an amount of arbitrage.

**Table 2:** Matrix of arbitrage amounts in different pairs of exchanges in 2019-01-13.

	kraken	bitbay	bitmarketlt	coindeal	dsx	cexio	bitlish	bitstamp	coinfalcon	coinfloor	coinmate	exmo
kraken	0	0	0.49	23.81	0	292.25	0	0	0	0	0	1389.67
dsx	0	0	0	9.63	0	78.12	0	0	0	0	0	0
bitbay	0	0	0	0	0	0	0	0	0	0	0	373.84
bitmarketlt	0	0	0	0.16	0	130.59	0	0	0	0	0	752.73
coindeal	0	0	0	0	0	0.68	0	0	0	0	0	1050.42
exmo	0	0	0	0	0	0	0	0	0	0	0	0
cexio	0	0	0	0	0	0	0	0	0	0	0	707.03
bitlish	0	0	0	0	0	252.28	0	0	0	0	0	1693.07
bitstamp	0	0	0.29	34.25	0	398.75	0	0	0	0	0	1354.16
coinfalcon	0	0	0.04	1.73	0	16.32	0	0	0	0	0	500.91
coinfloor	0	0	0	0	0	0	0	0	0	0	0	0
coinmate	0	0	0	0	0	0	0	0	0	0	0	0
quoinex	0	0	0	0	0	0	0	0	0	0	0	0

**Fig. 1:**
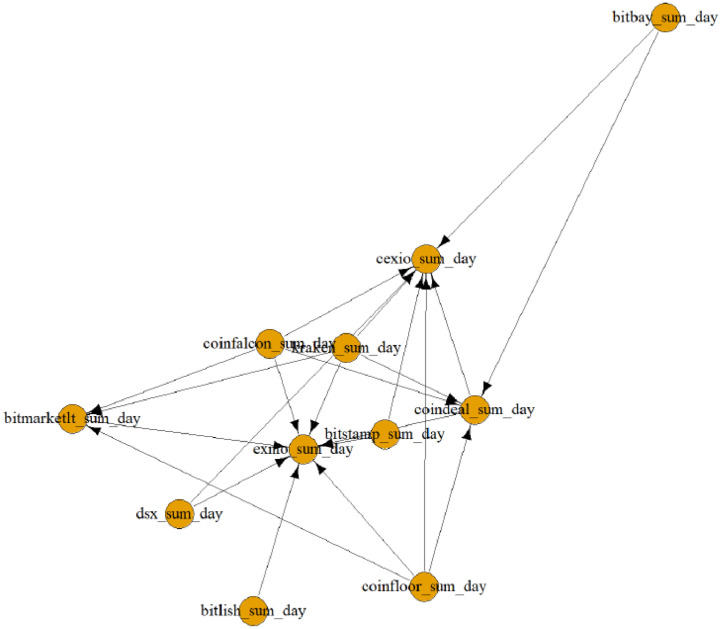
Graph of cryptocurrency exchanges’ network for 2019-01-13.

Matrices such as the one given in Table 2 were converted to graphs for each day and the network was presented in 455 different figures (see Fig. 1). All figures are given in the Mendeley Data repository.

## 2 Experimental Design, Materials and Methods

We received a large dataset which we had to prepare for the analysis (Table 3). As we introduced before, this dataset serves for various purposes. However, to reach our goal, we only needed data of arbitrage amounts and names of exchanges. We used dplyr package in R to filter only relevant data and to split timestamp in a required format for further data grouping. Sample of data which were further used is given in Table 4.

**Table 3:** Sample of raw dataset.

ID	arb_timestamp	arb_exch1	arb_xch2	arb_ticker	arb_prc	arb_amount	arb_quantity	best_sell_price	best_buy_price	balance_iat	balance_crypto
1	01/01/2019	bitstamp	cexio	BTCEUR	1.12	49.65	1.36	3228.29	3281	0	0
2	01/01/2019	bitstamp	therock	BTCEUR	0.30	0.14	0.02	3228.29	3252.68	0	0
3	01/01/2019	bitstamp	exmo	BTCEUR	3.93	1.27	0.01	3228.29	3370.57	0	0.02
4	01/01/2019	bitstamp	coinfalcon	BTCEUR	0.22	0.06	0.01	3228.29	3250	0	0.00
5	01/01/2019	bitstamp	coindeal	BTCEUR	0.30	2.08	0.21	3228.29	3259.20	0	0

**Table 4:** Sample of filtered dataset with only significant information for our empirical research.

ID	Year	Month	Day	time	arb_exch1	arb_exch2	arb_amount
1	2019	1	1	00:00:31	bitstamp	cexio	49.65
2	2019	1	1	00:00:31	bitstamp	therock	0.14
3	2019	1	1	00:00:31	bitstamp	exmo	1.27
4	2019	1	1	00:00:31	bitstamp	coinfalcon	0.06
5	2019	1	1	00:00:31	bitstamp	coindeal	2.08

First of all, we used plyr package to split data by “Exchange 2”. The process of generating the dataset we used for matrices creation is given by the points below:

1 We took data of each exchange, assigned required data format and using dpyr package grouped values by date and by “Exchange 1”.
2 Using reshape2 package we changed the shape of our dataset to suitable one for the analysis.
3 We grouped data again by date to calculated the amount of the arbitrage in each exchange for every day.
4 We merged these values to show how much it could be earned each day if Bitcoin was bought in “Exchange 1” and simultaneously sold to specific “Exchange 2”.

We repeated these 1–4 steps for every exchange (“Exchange 2”).

In the example given in Table 5 we present a sample of data showing how much it could be earned by buying Bitcoin in each exchange and simultaneously selling it to “Exchange 2” which is kraken in this case. In Table 5 ID represents days, so number 1 means 2019-01-01, number 2 means 2019-01-02 and so on.

**Table 5:** Sample of filtered dataset by kraken exchange.

ID	exmo	bitlish	bitmarketlt	bitstamp	cexio	coinfalcon	coinfloor	coinmate	coindeal	dsx	bitbay	quoinex	exch2
1	0	0	0	0	0	0	0	0	1356.61	0	0	0	kraken
2	0	36.74	65.98	0	0	0	0	0	34719.82	0	0	0	kraken
3	0	0	1.42	0	0	0	0	0	1338.52	0	0	0	kraken
4	0	5.35	0	0	0	0	0	0	50.89	60.68	0	0	kraken
5	0	0	9.03	0	0	0.95	0	0	378.48	1.87	0	0	kraken

After all these steps, we had 13 datasets for each exchange we're interested in. We merged them to get matrices for each day and finally, we created 455 matrices for the period from 2019-01-01 to 2020-04-01. Example of matrix for 2019-01-13 is given in Table 2.

## Ethics Statements

The work does not involve the subject of humans, animals, or data from social media platforms.

## CRediT Author Statement

**Rasa Bruzgė**: Methodology, Software, Data curation, Writing - Original draft preparation, Writing- Reviewing and Editing, Visualization. **Alfreda Šapkauskienė**: Conceptualization, Writing - Reviewing and Editing, Supervision.

## Declaration of Competing Interest

The authors declare that they have no known competing financial interests or personal relationships which have, or could be perceived to have, influenced the work reported in this article.
